# BCMA-targeted bispecific antibodies in relapsed/refractory multiple myeloma: three approvals, many questions

**DOI:** 10.1093/oncolo/oyag165

**Published:** 2026-04-28

**Authors:** Samer Al Hadidi, Adeel M Khan, Aimaz Afrough, Larry D Anderson

**Affiliations:** Myeloma, Waldenstrom’s, and Amyloidosis Program, Section of Hematologic Malignancies and Cellular Therapy, Simmons Comprehensive Cancer Center, UT Southwestern Medical Center, Dallas, TX 75390-8565, United States; Myeloma, Waldenstrom’s, and Amyloidosis Program, Section of Hematologic Malignancies and Cellular Therapy, Simmons Comprehensive Cancer Center, UT Southwestern Medical Center, Dallas, TX 75390-8565, United States; Myeloma, Waldenstrom’s, and Amyloidosis Program, Section of Hematologic Malignancies and Cellular Therapy, Simmons Comprehensive Cancer Center, UT Southwestern Medical Center, Dallas, TX 75390-8565, United States; Myeloma, Waldenstrom’s, and Amyloidosis Program, Section of Hematologic Malignancies and Cellular Therapy, Simmons Comprehensive Cancer Center, UT Southwestern Medical Center, Dallas, TX 75390-8565, United States

## Introduction

The approval of three B-cell maturation antigen (BCMA)-directed T-cell engaging bispecific antibodies (BsAbs)—teclistamab, elranatamab, and linvoseltamab—marks a significant moment in the treatment landscape for heavily pretreated multiple myeloma (MM). All three agents received accelerated FDA approval based on single-arm trials demonstrating high response rates in patients who had exhausted standard therapeutic options, including proteasome inhibitors, immunomodulatory agents, and anti-CD38 monoclonal antibodies.^1–3^ However, the inherent limitations of single-arm study designs, combined with differences in patient populations, trial conduct timelines, and outcome assessments, make direct comparisons between products impossible.

## The approved agents: similarities in indication, differences in detail

Teclistamab received initial FDA approval in 2022, followed by elranatamab in 2023 and most recently linvoseltamab in July 2025. All three share a common molecular target and mechanism—BsAbs bridging BCMA on malignant plasma cells with CD3 on T-cells to induce tumor cell lysis. However, differences in molecular structure (IgG4-PAA for teclistamab, IgG2Δa for elranatamab, and IgG4 for linvoseltamab), route of administration (subcutaneous for teclistamab and elranatamab versus intravenous for linvoseltamab), and dosing strategies reflect distinct developmental approaches.

## Trial design and patient populations: apples and oranges

The pivotal trials supporting these approvals—MajesTEC-1 (teclistamab, *N* = 110),^1^ MagnetisMM-3 Cohort A (elranatamab, *N* = 97),^2^ and LINKER-MM1 (linvoseltamab, *N* = 80)^3^—enrolled patients with broadly similar eligibility criteria requiring ≥3 prior lines of therapy or triple-class refractory status. However, there are meaningful heterogeneity that precludes direct comparison (Table 1).

**Table 1:** Baseline patient characteristics and efficacy outcomes.

Characteristic/outcome	Teclistamab (MajesTEC-1)	Elranatamab (MagnetisMM-3)	Linvoseltamab (LINKER-MM1)
**Baseline characteristics**			
** Median age (range)**	64 years (33-84)	69 years (46-89)	71 years (37-83)
** Age ≥75 years**	15%	18.6%	30%
** Male**	58%	52%	64%
** High-risk cytogenetics**	25%	22.7%	40%
** Extramedullary disease**	17%	34.0%	18%
** Triple-class refractory**	76%	96.9%	79%
** Median prior lines (range)**	5 (2-14)	5 (2-14)	5 (4-13)
**Efficacy outcomes**			
** Overall response rate (ORR)**	61.8%	57.7%	70%
** Complete response or better (≥CR)**	28.2%	25.8%	45%
** Very good partial response (VGPR)**	29.1%	25.8%	19%
** Partial response (PR)**	4.5%	6.2%	6%
** Median time to response (mo)**	1.2	1.22	0.95
** Median duration of response (mo)**	24	17.1	Not reached
** DOR rate at 9 months**	66.5%	82.3%	72%
** Median follow-up (mo)**	7.4	10.2	11.3

Patient demographics differed substantially.^4–6^ The median age ranged from 64 years in MajesTEC-1 to 71 years in LINKER-MM1, with varying proportions of patients ≥75 years (15% vs 18.6% vs 30%, respectively). Racial and ethnic composition also varied, with Asian patients representing 2% in MajesTEC-1, 13% in MagnetisMM-3, and 13% in LINKER-MM1, while Black or African American patients comprised 13%, 5.2%, and 14%, respectively. These demographic differences may influence both efficacy and toxicity outcomes but cannot be adjusted for in the absence of randomized comparisons.

Disease characteristics at baseline showed significant variation. High-risk cytogenetics were present in 25%, 22.7%, and 40% of patients, respectively. Extramedullary disease, a poor prognostic feature, was documented in 17%, 34%, and 18%. The proportion of patients with triple-class refractory disease—arguably the most treatment-resistant disease biology—ranged from 76% to 96.9%. These differences in disease biology and treatment refractoriness likely influenced response rates but their impact cannot be quantified without head-to-head comparisons.

## Efficacy: similar response rates with different follow-up

Overall response rates (ORR) were remarkably consistent: 61.8% for teclistamab, 57.7% for elranatamab, and 70% for linvoseltamab. Complete response (CR) or better rates ranged from 25.8% to 45%. While linvoseltamab showed the numerically highest ORR and CR rate, these differences occurred in the context of different patient populations, trial designs, and assessment methodologies. The overlapping 95% confidence intervals for ORR (52.1%-70.9%, 47.3%-67.7%, and 59%-80%, respectively) underscore the statistical uncertainty around point estimates from single-arm trials.

Duration of response data, though encouraging across all three agents with median DOR not reached, comes with the caveat of differing follow-up times for the initial reports (7.4-, 10.2-, and 11.3-months median follow-up among responders, respectively). The DOR rates at 9 months ranged from 66.5% to 82.3%.^4–6^ Longer-term follow-up data have emerged for two of these agents, providing important perspective on the durability of responses. For elranatamab, extended follow-up from MagnetisMM-3 (median 28.4 months) demonstrated a median progression-free survival (PFS) of 17.2 months and a median overall survival (OS) of 24.6 months in BCMA-naive patients.^7^ For teclistamab, updated analysis from MajesTEC-1 with 30.4 months median follow-up showed a median PFS of 11.4 months and median OS of 22.2 months in the overall population.^8^

## Safety: class effects versus agent-specific concerns

All three BCMA BsAbs share a common toxicity profile dominated by cytokine release syndrome (CRS), neurologic toxicity including immune effector cell-associated neurotoxicity syndrome (ICANS), infections, and cytopenias. However, the frequency and severity of these events varied across trials (Table 2).

**Table 2:** Safety profile and adverse events.

Adverse event	Teclistamab	Elranatamab	Linvoseltamab
**Cytokine release syndrome**			
** Any grade CRS**	72%	58%	46%
** Grade 1**	50%	44%	35%
** Grade 2**	21%	14%	10%
** Grade 3**	0.6%	0.5%	0.9%
** Grade 4**	0%	0%	0%
** Recurrent CRS**	33%	13%	20%
** Median time to onset**	2 days	2 days	11 hours
** Median duration**	2 days	2 days	15 hours
**Neurologic toxicity**			
** Any grade neurologic toxicity**	57%	59%	54%
** Grade 3-4 neurologic toxicity**	2.4%	7%	8%
** ICANS (any grade)**	6%	3.3%	8%
** ICANS grade 3-4**	1 Grade 4 event^a^	Not specified	2.6%
** Median time to onset (d)**	4 (2-8)	3 (1-4)	1 (1-4)
** Median duration (d)**	3 (1-20)	2 (1-18)	2 (1-11)
**Infections**			
** Any serious infections**	30%	42%	42%
** Grade 3-4 infections**	35%	31%	38%
** Fatal infections**	4.2%	7%	4%
** Pneumonia (any grade)**	24%	32%	28%
** Pneumonia (grade 3-4)**	15%	19%	21%
** Upper respiratory infection**	26% (2.4% Grade 3-4)	36% (4.9% Grade 3-4)	35% (6% Grade 3-4)
**Hematologic abnormalities**			
** Lymphocyte count decreased (grade 3-4)**	84%	84%	92%
** Neutrophil count decreased (grade 3-4)**	56%	51%	47%
** Hemoglobin decreased (grade 3-4)**	33%	43%	42%
** Platelet count decreased (grade 3-4)**	22%	32%	19%

a With longer follow-up, teclistamab showed one Grade 4 seizure and one fatal Guillain-Barré syndrome case.

**Cytokine release syndrome**: Any grade CRS occurred in 46% (linvoseltamab), 58% (elranatamab), and 72% (teclistamab) of patients. The higher rate with teclistamab may reflect earlier trial conduct with evolving CRS recognition and management, differences in CRS grading implementation, or true biological differences. Importantly, Grade 3-4 CRS remained rare (0.6%-0.9%) across all agents, and no Grade 4 events occurred. The median time to onset (11 h to 2 d) and duration (15 h to 2 d) were generally consistent. Recurrent CRS rates varied substantially (13%-33%), with teclistamab showing the highest rate.^4–6^

**Neurologic toxicity**: Overall neurologic toxicity occurred in 54%-59% of patients across the three agents. ICANS, a more specific concern, occurred in 3.3% (elranatamab), 6% (teclistamab), and 8% (linvoseltamab). With longer follow-up, teclistamab showed one Grade 4 seizure and one fatal Guillain-Barré syndrome case, highlighting the potential for delayed severe neurologic events. However, no Parkinsonism or cranial nerve palsies were reported.

**Infections**: Serious infections occurred in 30%-42% of patients, with Grade 3-4 infections in 31%-38%, and fatal infections in 4%-7%. Pneumonia was the most common serious infection (13%-25% across agents).^9^

**Hematologic toxicity**: All three agents caused high rates of Grade 3-4 lymphopenia (84%-92%), neutropenia (47%-56%), and anemia (33%-43%). These cytopenias reflect both disease-related marrow dysfunction and treatment-related T-cell and B-cell engagement.

## The unanswerable question: which agent is best?

In the absence of head-to-head randomized trials, declaring one agent superior to another is scientifically untenable. The differences in efficacy and safety profiles observed across trials could reflect: (1) true biological differences in antibody structure, affinity, or pharmacodynamics; (2) patient population heterogeneity in baseline characteristics and disease biology; (3) temporal factors including evolution of supportive care and trial conduct during different pandemic phases; (4) assessment differences in response evaluation and adverse event attribution; or (5) institutional factors including center experience with BsAbs.

Single-arm trial designs, while necessary for expedited drug development in heavily pretreated populations with limited options, cannot account for these confounding variables. Cross-trial comparisons are hypothesis-generating at best and misleading at worst.

## Clinical decision-making in the absence of comparative data

How should clinicians choose among these three agents? Several practical considerations may inform decision-making:

**Route of administration preferences**: Some patients or institutions may prefer subcutaneous delivery (teclistamab or elranatamab) versus intravenous delivery (linvoseltamab) based on venous access, patient convenience, or resource availability.

**Dosing schedule**: The possibility of less frequent dosing (every 4 weeks) may influence long-term treatment selection for patients achieving deep responses.

**Institutional experience**: Centers with established infrastructure and experience managing specific agents may achieve better outcomes through protocol adherence and rapid toxicity recognition.

**Economic considerations and pricing**: In the absence of clear efficacy or safety advantages, financial factors may increasingly drive treatment selection. However, selection based solely on lowest acquisition cost, while economically attractive, oversimplifies complex clinical decision-making and may not account for the total cost of care, including management of adverse events, hospitalization requirements, and long-term outcomes. Moreover, equal access to all three agents should be maintained to preserve clinical decision-making flexibility, as individual patient factors may favor one agent over another despite similar population-level outcomes. Transparent pricing information and comparative cost-effectiveness analyses will be essential to inform value-based decisions without compromising patient care.

## How I choose in my practice

In the absence of definitive comparative data, we prioritize three practical factors: cost, dosing frequency, and route of administration. With pricing ranging from $18 000-22 000 per cycle and no clear clinical superiority among these agents, we select the most cost-effective option for both the hospital and patient. We prefer agents offering less frequent administration schedules (every 4 weeks when possible) to reduce healthcare visits and costs; all three agents—elranatamab, teclistamab, and linvoseltamab—allow step-down to an every-4-week dosing schedule in patients achieving deep responses (VGPR or better), making this a shared advantage across the class rather than a differentiating factor. When these factors align, the choice is clear; when they conflict, we engage in shared decision-making that emphasizes clinical equivalence while weighing cost and convenience.

## The path forward

All three BCMA BsAbs have received accelerated approval contingent upon confirmatory trials demonstrating clinical benefit. These ongoing randomized trials will provide higher-quality evidence for efficacy, though head-to-head comparisons remain unlikely unless conducted through cooperative groups. The field would benefit from: (1) standardized outcome assessments including minimal residual disease evaluation and quality-of-life measures; (2) longer-term follow-up data to assess durability of responses and late toxicities; (3) biomarker studies to identify predictors of response and toxicity; (4) sequencing studies to define optimal integration with other therapies; and (5) real-world effectiveness studies to complement controlled trial data.

## Conclusion

The approval of three BCMA BsAbs represents meaningful progress for patients with heavily pretreated MM. All three agents demonstrate excellent activity with manageable, though significant, toxicity profiles. However, the single-arm trial designs, patient population differences, and temporal factors preclude definitive conclusions about comparative efficacy or safety. While economic theory suggests that multiple approved agents should create market competition and reduce costs through pricing pressure, the oncology pharmaceutical market has not consistently demonstrated such price reductions following competitive approvals. Nevertheless, the availability of three agents provides healthcare systems with negotiating leverage and ensures therapeutic alternatives if supply chain or access issues arise with any single agent.

## Author contributions

Samer Al Hadidi (Conceptualization, Formal analysis, Writing—original draft, Writing—review & editing), Adeel M. Khan (Writing—review & editing), Aimaz Afrough (Writing—review & editing), and Larry D. Anderson (Conceptualization)

## Funding

None declared.

## Conflicts of interest

S.A. reports receiving consulting fees from Janssen, Legend Biotech and Sanofi. A.M.K. reports Research: Sanofi, Janssen. X.W.: Employment: Flatiron Health, Inc. (independent subsidiary of the Roche Group), stock ownership, Roche. A.A. reports Advisory role for Karyopharm, BMS, Sanofi, Johnson & Johnson, Pfizer, and research funding from Abbvie, Adaptive Biotech, K36-therapeutics, for Johnson & Johnson, Regeneron Pharmaceuticals. L.D.A. reports Consulting fees and Advisory Board activity from Janssen (Johnson & Johnson), Celgene, BMS, Amgen, GSK, AbbVie, Beigene, Karyopharm, Pfizer, Cellectar, Sanofi, Prothena.

## Data Availability

The manuscript cited publicly available data with no original data to provide.
